# In Vitro and In Silico Evaluation of *Syzygium aromaticum* Essential Oil: Effects on Mitochondrial Function and Cytotoxic Potential Against Cancer Cells

**DOI:** 10.3390/plants13233443

**Published:** 2024-12-08

**Authors:** Andreea Munteanu, Armand Gogulescu, Codruța Șoica, Alexandra Mioc, Marius Mioc, Andreea Milan, Alexandra Teodora Lukinich-Gruia, Maria-Alexandra Pricop, Calin Jianu, Christian Banciu, Roxana Racoviceanu

**Affiliations:** 1 Department of Internal Medicine IV, Faculty of Medicine, “Victor Babes” University of Medicine and Pharmacy, 2 Eftimie Murgu, 300041 Timisoara, Romania; munteanu.andreea@umft.ro (A.M.); banciu.christian@umft.ro (C.B.); 2 Department XVI: Balneology, Medical Rehabilitation and Rheumatology, “Victor Babes” University of Medicine and Pharmacy, 2 Eftimie Murgu, 300041 Timisoara, Romania; 3 Department of Pharmacology-Pharmacotherapy, Faculty of Pharmacy, Victor Babes University of Medicine and Pharmacy, Eftimie Murgu Square, No. 2, 300041 Timisoara, Romania; codrutasoica@umft.ro (C.Ș.); alexandra.mioc@umft.ro (A.M.); 4 Research Centre for Pharmaco-Toxicological Evaluation, Victor Babes University of Medicine and Pharmacy, Eftimie Murgu Square, No. 2, 300041 Timisoara, Romania; marius.mioc@umft.ro (M.M.); andreea.milan@umft.ro (A.M.); babuta.roxana@umft.ro (R.R.); 5 Department of Pharmaceutical Chemistry, Faculty of Pharmacy, Victor Babes University of Medicine and Pharmacy, Eftimie Murgu Square, No. 2, 300041 Timisoara, Romania; 6 OncoGen Centre, Clinical County Hospital “Pius Branzeu”, Blvd. Liviu Rebreanu 156, 300723 Timisoara, Romania; alexandra.gruia@hosptm.ro (A.T.L.-G.); alexandra.pricop@oncogen.ro (M.-A.P.); 7 Department of Applied Chemistry and Environmental Engineering and Inorganic Compounds, Faculty of Industrial Chemistry, Biotechnology and Environmental Engineering, Polytechnic University of Timisoara, Vasile Pârvan 6, 300223 Timisoara, Romania; 8 Faculty of Food Engineering, Banat’s University of Agricultural Sciences and Veterinary Medicine “King Michael I of Romania”Timisoara, Calea Aradului 119, 300645 Timisoara, Romania; calin.jianu@gmail.com

**Keywords:** clove (*Syzygium aromaticum* L.), essential oil, anticancer activity, natural compounds, molecular docking

## Abstract

The current study proposes the in vitro and in silico anticancer evaluation of clove (*Syzygium aromaticum* L.) essential oil (CEO). The steam hydrodistillation method used yielded 10.7% (wt) CEO. GC-MS analysis revealed that the obtained oil is rich in eugenol (75%), β-caryophyllene (20%), and α- caryophyllene (2.8%) and also contains several other minor components accounting for approximately 1.5%. The DPPH-based scavenging antioxidant activity was assessed for the obtained CEO, exhibiting an IC_50_ value of 158 μg/mL. The cytotoxic effects of CEO, its major component eugenol, and CEO solubilized with Tween-20 and PEG-400 were tested against both noncancerous HaCaT cells and HT-29 human colorectal adenocarcinoma, RPMI-7951 melanoma, A431 skin carcinoma, and NCI-H460 non-small lung cancer cells, using the Alamar Blue and LDH assay after 48 h treatment. The Tween-20 and PEG-400 CEO formulations, at 200 μg/mL, recorded the highest cytotoxic and selective effects against RPMI-7951 (72.75% and 71.56%), HT-29 (71.51% and 45.43%), and A431 cells (61.62% and 59.65%). Furthermore, CEO disrupted mitochondrial function and uncoupled oxidative phosphorylation. This effect was more potent for the CEO against the RPMI-7951 and HT-29 cells, whereas for the other two tested cell lines, a more potent inhibition of mitochondrial function was attributed to eugenol. The present study is the first to specifically investigate the effects of CEO and Tween-20 and PEG-400 CEO formulations on the mitochondrial function of RPMI-7951, HT-29, A431, and NCI-H460 cancer cell lines using high-resolution respirometry, providing novel insights into their impact on mitochondrial respiration and bioenergetics in cancer cells. The results obtained may explain the increased ROS production observed in cancer cell lines treated with eugenol and CEO. Molecular docking identified potential protein targets, related to the CEO anticancer activity, in the form of PI3Kα, where the highest active theoretical inhibitor was calamenene (−7.5 kcal/mol). Docking results also showed that calamenene was the overall most active theoretical inhibitor for all docked proteins and indicated a potential presence of synergistic effects among all CEO constituents.

## 1. Introduction

Despite current efforts to diminish its risk factors as well as prevalence, cancer still stands as the leading cause of death worldwide [1]. The current treatment strategy involves a combination of chemotherapy, radiotherapy, and/or surgical resection [2]. Even though current treatment protocols are updated continuously in order to increase patients’ survival rates, conventional chemotherapy and radiotherapy cause significant side effects due to their non-selective DNA damage inflicted in healthy tissues and cells [3]; they may also induce cancer cell resistance to treatment. Furthermore, the poor pharmacokinetic characteristics of antineoplastic drugs lead to the imperative need to develop novel anticancer compounds with optimized pharmacological properties [4]. 

The use of natural products as treatment against various pathologies stands as the most significant precursor of modern medicine [5]. Natural compounds play a pivotal role in cancer management due to the wide range of substances of natural origin or derived from natural products that are currently used in therapy [6]. Essential oils (EOs) can be extracted from different plants and exhibit a complex composition that may display more than 300 different compounds [7]; they have gained significant importance in the last years due to their popularity in different industries, including their use as natural treatments for various diseases [8]. For example, essential oils have been extensively used to alleviate symptoms associated with cardiovascular diseases, sleep and stress disorders, or Alzheimer’s disease [9]. The EOs’ investigation as anticancer agents is relatively new, yet several molecular mechanisms including apoptosis through both intrinsic and extrinsic pathways were already identified as involved in exerting anticancer effects [10]. Moreover, even though essential oils cannot replace entirely conventional chemotherapy, they are able to mitigate some of its adverse effects, thus increasing the patient’s life quality [11]; additionally, essential oils are able to increase the efficacy of associated chemotherapy drugs [10]. *Eugenia caryophyllata*, commonly known as clove, is a spice belonging to the Myrtaceae family that has been employed as a food preservative and also used for a plethora of therapeutic purposes such as antimicrobial, antioxidant and anti-inflammatory [12]. Several scientific reports demonstrated that clove contains 15–20% essential oil exhibiting high amounts of phenolic compounds with numerous biological effects such as antibacterial, antioxidant, and antifungal [13]. Clove essential oil was found active as an antiproliferative agent against HTh-7 thyroid cancer cells [14], HT-29 and Caco-2 colon cancer cells [15,16], HepG2 liver, and MCF-7 breast cancer cells [17] as well as several other cancer cell lines; it is noteworthy to emphasize that the essential oil exerted stronger anticancer effects compared to its pure major constituents such as eugenol, thus revealing a potential synergistic association with minor compounds [18]. Despite the pharmacological potential of clove essential oil (CEO), its therapeutic application is hampered by its high volatility, low water solubility, and sensitivity to light [19]. To overcome these setbacks while also avoiding the use of organic solvents, EOs can be emulsified into oil-in-water emulsions by employing surfactants such as polyethylene glycol (PEG) or Tween [20]. 

The main objective of our current study was to assess the anticancer potential of CEO dispersed in PEG 400 and Tween 20 against several cancer cell lines (HT-29 human colorectal adenocarcinoma, RPMI-7951 melanoma, A431 skin carcinoma, NCI-H460 non-small lung cancer) as well as against human immortalized keratinocyte (HaCaT) cells and to conduct a docking-based in silico identification of the potential biological targets of the main compounds in CEO. 

## 2. Results

### 2.1. Chemical Composition of CEO

The steam hydrodistillation of the dried ground cloves yielded 10.7% (wt) of a pale-yellow oil with an intense characteristic eugenol odor. The GC-MS analysis of the clove essential oil (CEO) revealed the presence of eugenol (75.26%), β-caryophyllene (20.22%), and α-caryophyllene (2.84%) as major components, accompanied by several other minor compounds, as shown in Table 1.

### 2.2. Determination of the Antioxidant Activity of CEO

**Figure 1:**
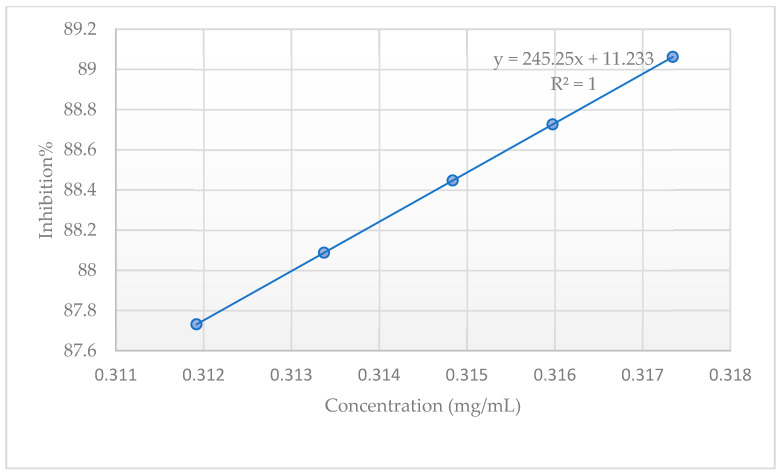
DPPH inhibitory activity of CEO.

The antioxidant scavenging activity of CEO was determined by the 2,2-diphenyl-1-picrylhydrazyl (DPPH) radical assay using ascorbic acid (AA) and butylated hydroxyanisole (BHA) as positive controls. The results are available in Table 2. While the IC_50_ recorded value for CEO is 158 µg/mL, a 90% inhibition of DPPH activity can be reached at a concentration of approximately 320 µg/mL of essential oil. The DPPH titration curve is depicted in Figure 1.

### 2.3. Evaluation of CEO Cytotoxic Effect

#### 2.3.1. CEO Effect on Cell Viability

**Figure 2:**
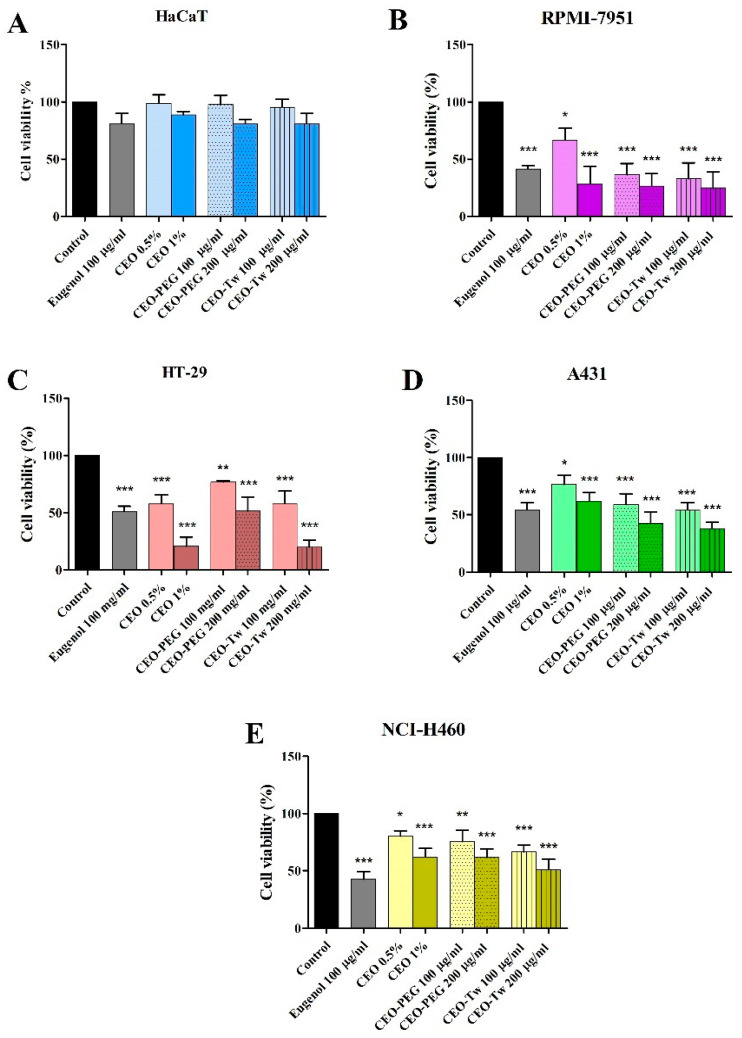
Cell viability after 48 h treatment with eugenol 100 μg/mL, CEO dispersed directly in the cell culture media (CEO 0.5% and 1%), CEO mixed with emulsifying agents PEG-400 and Tween 20 (CEO-PEG and CEO-Tw 100 μg/mL, 200 μg/mL) on HaCaT (**A**), RPMI-7951 (**B**), HT-29 (**C**), A431 (**D**), and NCI-H460 (**E**) cells. The results are expressed as viability percentages compared to the control group, considered 100%. The data represent the mean values ± SD of three independent experiments performed in triplicate (* *p* < 0.05, ** *p* < 0.01, and *** *p* < 0.001).

The effect of eugenol (100 μg/mL), CEO (0.5% and 1%), CEO-PEG (100 and 200 μg/mL), and CEO-Tw (100 and 200 μg/mL) on normal HaCaT cells and RPMI-7951, HT-29, A341, and NCI-H460 was assessed using the Alamar Blue assay after 48 h treatment; the results are presented in Figure 2. No significant cell viability changes were observed when PEG-400 and Tween 20 were tested independently, without CEO, on all the cell lines used in this study. Neither of the compounds decreased in a statistically significant manner the cell viability of normal HaCaT cells. The highest degree of cell viability inhibition observed in malignant melanoma RPMI-7951 cells was achieved after the treatment with the highest concentrations of CEO mixed with the emulsifying agents Tween 20 and PEG-400; this was followed by the effect of the highest tested concentration of CEO dispersed directly in the cell culture media, then by the lower concentrations of CEO combined with solubilizing agents, eugenol, and finally, by the lower concentrations of CEO dispersed directly in the cell culture media. The specific results given as reduced viability percentages were as follows: 24.87% (CEO-Tw 200 μg/mL), 26.52% (CEO-PEG 200 μg/mL), 28.48% (CEO 1%), 32.92% (CEO-Tw 100 μg/mL), 36.47% (CEO-PEG 100 μg/mL), 41.32% (eugenol), and 66.49% (CEO 0.5%). On HT-29, eugenol (100 μg/mL), CEO (0.5% and 1%), CEO-PEG (100 and 200 μg/mL), and CEO-Tw (100 and 200 μg/mL) decreased cell viability (%) vs. control (considered 100%), as follows: 51.23%, 57.87%, 20.69%, 76.97%, 51.56%, 58.17%, and 19.96%, respectively. On the epidermoid carcinoma cell line, A431, the highest concentrations of CEO-PEG (42.27%) and CEO-Tw (39.36%) were able to decrease significantly cell viability vs. control (100%) and also vs. eugenol (54.32%). CEO 0.5% (76.68%), CEO 1% (61.89%), CEO-PEG 100 μg/mL (58.9%), and CEO-Tw 100 μg/mL (54.34%) decreased cell viability in a significant manner vs. control; however, their effect was as strong as the effect recorded for eugenol (54.32%). On the NCI-H460 cell line, the strongest cytotoxic effect was recorded for eugenol (42.57%), followed by CEO-Tw 200 μg/mL (50.95%), CEO-PEG 200 μg/mL (61.70%), CEO 1% (62.12%), CEO-Tw 100 μg/mL (66.31%), CEO-PEG 100 μg/mL (75.59%), and CEO 0.5% (80.26%).

#### 2.3.2. Determination of CEO Cytotoxic Potential by Lactate Dehydrogenase (LDH) Release

**Figure 3:**
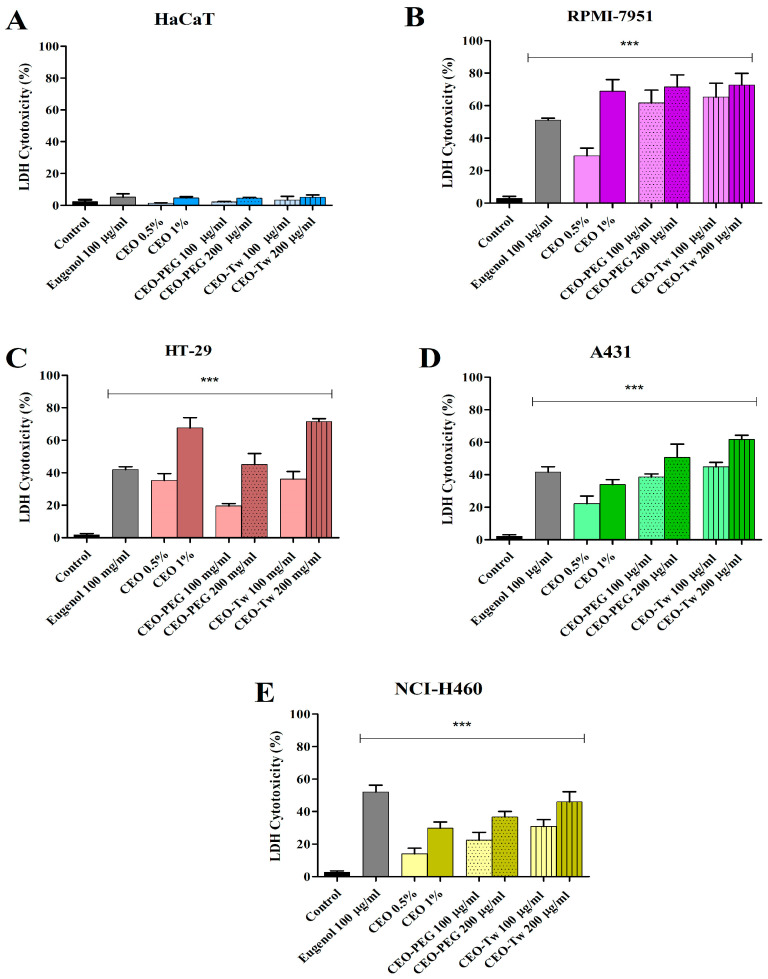
Lactate dehydrogenase (LDH) release percentages in HaCaT (**A**), RPMI-7951 (**B**), HT-29 (**C**), A341 (**D**), and NCI-H460 (**E**) cells exposed for 48 h to eugenol (100 μg/mL), CEO (0.5%, 1%), CEO-PEG (100 and 200 μg/mL), and CEO-Tw (100 and 200 μg/mL). The results were expressed as means ± standard deviation of three experiments performed in triplicate (*** *p* < 0.001).

The cytotoxic effect of eugenol (100 μg/mL), CEO (0.5%, 1%), CEO-PEG (100 and 200 μg/mL), and CEO-Tw (100 and 200 μg/mL) on HaCaT, RPMI-7951, HT-29, A341, and NCI-H460 cells was evaluated using the LDH assay. Following 48 h of treatment, the cytotoxicity evaluation revealed that all the tested compounds were able to induce a significant cytotoxic effect on all cancer cell lines used in this study at all concentrations tested (Figure 3). No significant cytotoxic effect was recorded on the normal HaCaT cell line (Figure 3). The Tween-20 and PEG-400 CEO formulations, at 200 μg/mL, recorded the highest cytotoxic and selective effects against RPMI-7951, HT-29, and A431 cell lines, compared with control. On RPMI-7951, CEO-Tw promoted LDH release up to 72.75%, CEO-PEG to 71.56%, and eugenol alone to 51.06%. On HT-29, CEO-Tw increased with 71.51% LDH release, CEO-PEG with 45.43%, and eugenol with 41.95%. On A431 cells, the obtained LDH release was as follows: CEO-Tw 61.62%, CEO-PEG 59.65%, and eugenol 41.50% (Figure 3).

### 2.4. Evaluation of CEO on ROS Production

**Figure 4:**
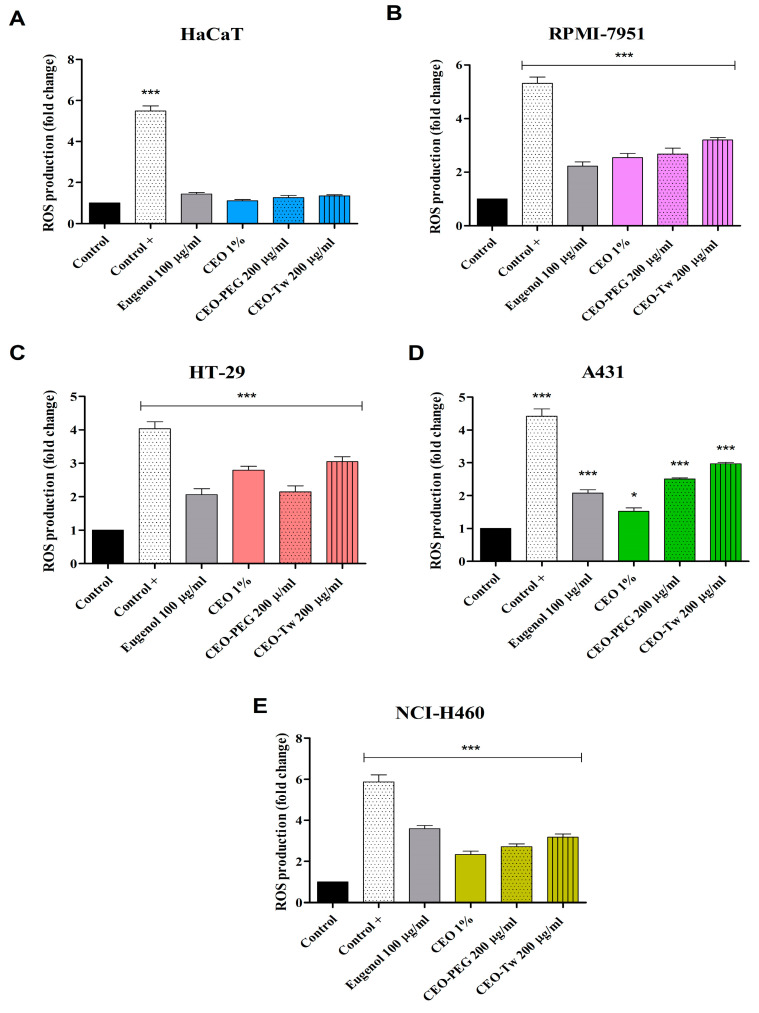
Assessment of ROS production in HaCaT (**A**), RPMI-7951 (**B**), HT-29 (**C**), A341 (**D**), and NCI-H460 (**E**) cells exposed for 48 h to eugenol (100 μg/mL), CEO (1%), CEO-PEG (200 μg/mL), and CEO-Tw (200 μg/mL). Tert-butyl hydroperoxide (TBHP) was used as a positive control. The data represent the results of three independent experiments and are presented as the mean ± S.D (* *p* < 0.05 and *** *p* < 0.001).

The investigation of the effect of eugenol (100 μg/mL), CEO (1%), and CEO formulated with PEG 400 and Tween 20 (200 μg/mL) on reactive oxygen species (ROS) production in HaCaT, RPMI-7951, HT-29, A341, and NCI-H460 cells was determined by DCFDA assay. Treatment with eugenol, simple CEO water dispersion, and PEG and Tween CEO formulations significantly increased ROS production in all the tested cancer cell lines whilst having no appreciable effect on normal HaCat cells (Figure 4A). The highest increase in ROS production was recorded for Tween 20 CEO formulation (200 μg/mL) in RPMI-7951, HT-29, and A341 cells, as follows: 3.23, 3.05, and 2.98 vs. control (1). The second highest increase in ROS production was obtained after the treatment with CEO-Tw formulation: 2.67, 2.14, and 2.52. The CEO formulation induced a higher increase in ROS production in these cell lines compared with eugenol alone, which resulted in fold changes of 2.23, 2.06, and 2.08. (Figure 4B–D). In NCI-H460 cells, the highest degree of ROS production increase was observed after the treatment with eugenol (3.59), followed by CEO-Tw (2.71) and CEO-PEG (3.18) vs. control (Figure 4E). 

### 2.5. Evaluation of CEO on Mitochondrial Function

**Figure 5:**
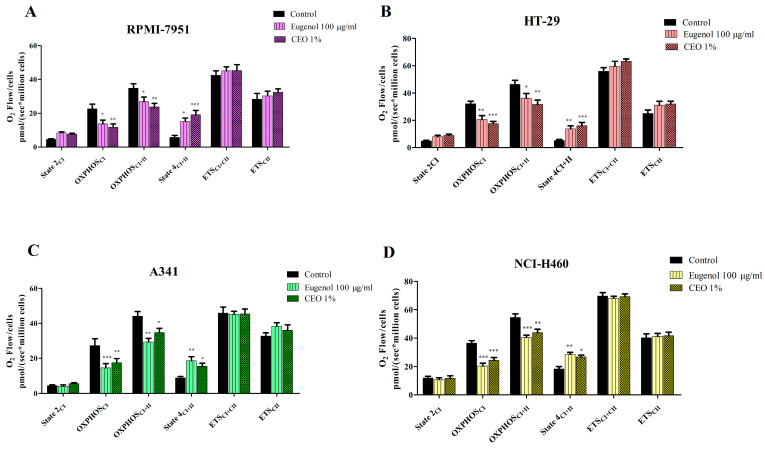
Mitochondrial respiratory rates of permeabilized RPMI-7951 (**A**), HT-29 (**B**), A341 (**C**), and NCI-H460 (**D**) cells after the treatment with 100 μg/mL eugenol and CEO 1%. Results are expressed as mean values ± SD of three independent experiments (* *p* < 0.05, ** *p* < 0.01, and *** *p* < 0.001).

The effect of eugenol and CEO on mitochondrial function of permeabilized RPMI-7951, HT-29, A341, and NCI-H460 cancer cells was assessed using high-resolution respirometry. The results showed that both compounds inhibit active respiration, dependent on both CI and CII, and also induce an uncoupling effect on all the tested cell lines (Figure 5). More precisely, eugenol and CEO 1% significantly decreased active respiration (OXPHOS_CI_ and OXPHOS_CI+II_) of RPMI-7951, HT-29, A341, and NCI-H460 vs. control (Table 3). Another noteworthy mention is the decrease in OXPHOS_CI_ vs. OXPHOS_CI+II_; it seems that both compounds inhibit to a higher degree OXPHOS_CI_ as compared with OXPHOS_CI+II_ (Table 3). When measuring the electron transport system’s maximum respiratory capacity (ETS_CI_ and ETS_CI+II_) after the treatment with eugenol and CEO 1%, we did not observe any significant changes compared to the control in all the cell lines studied. State 4 significantly increased vs. control in all cell lines after the treatment with eugenol and CEO 1%. Intriguingly, neither of the compounds had any significant effect on State 2. Taken together, the increase in proton leak (State 4) with the inhibition of OXPHOS (CI and CI+II) suggests that the compounds exert an uncoupling effect while also inhibiting active respiration. 

### 2.6. Molecular Docking

Molecular docking is a helpful computational tool that can be used to elucidate a possible protein-targeted action mechanism of a biologically active molecule [21,22]. For the present study, we used a molecular docking-based workflow to determine possible protein targets for the 10 CEO components, whose inhibition could be correlated with their in vitro anticancer cytotoxic activity. Therefore, we docked the CEO components against druggable protein targets that are usually overexpressed in various types of cancer and are correlated with increased cell proliferation and survivability; these protein targets were epidermal growth factor receptor 1 (EGFR1), vascular endothelial growth factor receptor 2 (VEGFR2), phosphatidylinositol 4,5-bisphosphate 3-kinase catalytic subunit alpha isoform (PI3Kα), dual specificity mitogen-activated protein kinase 1 (MEK1), protein kinase B (AKT/PKB), mammalian target of rapamycin (mTOR), apoptosis regulator Bcl-X (Bcl-XL), and apoptosis regulator Bcl-2 (Bcl-2). Docking scores of compounds **1**–**10** and each target’s native ligand (NLs), used as positive controls, are listed in Table 4.

**Figure 6:**
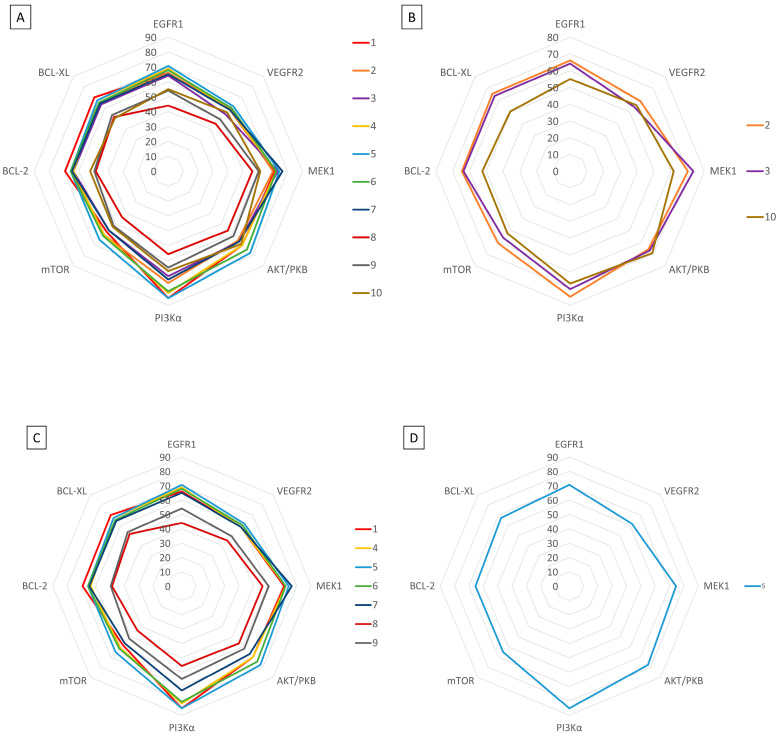
Docking score radar graphs corresponding to all docked components of the CEO (**A**), the major components (**B**), the minor components (**C**), and the overall highest theoretically active component, calamenene (**D**); the graph lines are plotted using percentage values of each compound’s docking score relative to their respective NL docking score.

**Figure 7:**
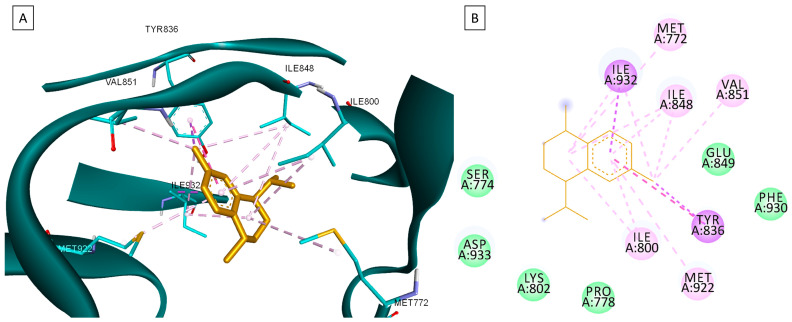
Compound 5 (Calamenene—orange) docked in the active site of PI3Kα (PDB ID: 6GVF), in 3D (**A**) and 2D (**B**) representation; formed hydrophobic interactions are depicted as purple dotted lines.

None of the docked compounds recorded lower binding affinities than the NLs, used as positive controls. However, in order to compare a possible cumulative effect of the ten compounds against a certain protein target, all recorded docking scores were calculated as a percentage of their respective NL’s docking score, after which these values were plotted as a radar graph where each corner represents one of the eight protein targets used. If the case, the graph should show the lines (representing the affinity values) oriented closer towards one or more proteins. Regarding the current case, the compounds’ affinity lines are drawn closer to PI3Kα (Figure 6A). We separated the first graph into two separate graphs depicting the major (compounds **2**, **3**, and **10**) and minor components (compounds **1** and **4**–**9**) to evaluate if this affinity trend holds for both cases. As observed in Figure 6B,C, the overall tendency towards PI3Kα is still present in both subcategories of CEO components. Compound **5** (calamenene) is the highest active theoretical inhibitor of PI3Kα apart from the NL (Figure 6D) and the overall most active theoretical inhibitor, ranking as the highest or second highest active docked compounds, apart from the NL, for all docked proteins. Calamenene is a bicyclic triterpene, thus containing no heteroatoms that are able to form hydrogen bonds with the neighboring amino acid residues within the binding site. Despite all this, calamenene is well bound in the Pi3K binding site, forming multiple hydrophobic interactions with several amino acid residues. Interacting amino acids include MET772, ILE800, TYR836, ILE848, VAL851, MET922, and ILE932. Formed interactions between calamenene and PI3Kα in 2D and 3D representation are depicted in Figure 7.

### 2.7. In Silico Pharmacokinetic and Toxicologic Profile Prediction for the CEO Components

For the purpose of our current work, we employed the SwissADME server to predict various pharmacokinetic parameters for all ten CEO components. The retrieved parameters were GI (gastro-intestinal) absorption, BBB (blood–brain barrier) permeant, Pgp (P-glycoprotein) substrate, CYP1A2 inhibitor, CYP2C19 inhibitor, CYP2C9 inhibitor, CYP2D6 inhibitor, and CYP3A4 inhibitor. The obtained results are available in Table 5.

Out of all analyzed components, four compounds including eugenol (compound **10**), the primary component of CEO, show a high predicted absorption rate from the GI tract. Most compounds can cross the blood–brain barrier with the exception of both caryophyllene isomers (compounds **2**, **3**) and calamenene (**5**). There is a high variability among these compounds towards CYP450 isoform inhibition. Only three minor components, namely 2,5,9-trimethylcycloundeca-4,8-dienone, 1,2-dimethylcyclohexane, and 3,4-dimethyl-3-cyclohexenylmethanal (compounds **6**, **8**, and **9**, respectively) are completely absent of any CYP450 predicted inhibition. None of the analyzed molecules are a substrate for P-glycoprotein (PGP).

For the predicted toxicology profile of the CEO companies, we used the PreADMET server to retrieve variables regarding potential rodent carcinogenicity (Carcino_Rat, Carcino_Mouse), crustaceans and small fish acute toxicity (daphnia_at, medaka_at, and minnow_at, respectively), as well as the inhibitory potential for the human Ether-à-go-go-Related Gene (hERG). The results are available in Table 6.

According to the obtained results, all CEO components show a predicted degree of rodent carcinogenicity, whether present solely for mice, rats, or both. This prediction is based on a model created “from the data of NTP (National Toxicology Program) and US FDA, which are the results of the in vivo carcinogenicity tests of mice and rats for 2 years”, as the server web page states [23]. It is unclear how dose-related this prediction model is. According to data acquired and based on Directive 92/32/EEC of the European Chemicals Law, substances considered hazardous to the aquatic environment are those with solubility values > 1 mg/mL. The remaining compounds that have solubilities below this threshold are categorized as having the potential to “cause long-term adverse effects in the aquatic environment” [24]. Regardless of the predicted aquatic animal model, all substances are relatively safe, falling into the <1 mg/mL solubility category. The last predicted toxicologic parameter is related to hERG inhibition. When administering drugs that inhibit hERG, which codes for the potassium channel involved in cardiac repolarization, the QT interval may lengthen, leading to Torsade de Pointes, a potentially lethal ventricular tachyarrhythmia [25]. In this regard, all ten CEO components show a medium to low risk of hERG-predicted inhibition.

## 3. Discussion

Essential oils are complex mixtures of volatile compounds extracted from aromatic plants where they are synthesized as secondary metabolites [26]. They act as main ingredients in aromatherapy, a holistic healing technique that promotes overall well-being and stands as an adjuvant in a wide range of pathologies, including cancer. Although relatively recently, research in the field has exposed the direct antiproliferative activity of essential oils against various cancer cell lines, thus emphasizing their complex biological effects. Clove oil is extracted from the flower buds of the *E. caryophyllata* tree [27] and has been used traditionally as a natural remedy against infectious and gastrointestinal disorders [28]. It is recognized as generally safe by the World Health Organization (WHO), which established the acceptable daily human intake able to reduce several health risks [29]. Clove oil was evaluated as an antiproliferative agent against cervical (HeLa), pancreatic (Panc), and colon (HCT) cancer cells, where it showed stronger biological effects than conventional chemotherapy (i.e., doxorubicin) [30]; it also inhibited five different human cancer cell lines (liver HepG2, breast MCF-7, prostate PC3, colon HCT116, and lung A549) with a potency similar to doxorubicin [31].

The therapeutic effects of clove essential oil have been attributed to the presence of several chemical components, mainly eugenol (more than 50%), β-caryophyllene, α-caryophyllene, and acetyl eugenol [32,33,34]. In our sample, GC-MS analysis revealed the presence of more than 75% eugenol and 20% beta-caryophyllene, accompanied by various trace amounts of minor polyphenolic constituents. Eugenol exhibited strong anticancer activity against numerous cancer cell lines such as colon, gastric, breast, prostate, skin, melanoma, and leukemia, where it inhibited tumor development and progression by generating reactive oxygen species (ROS), cytochrome-c and LDH release, apoptosis, and genotoxic effects [13,35].

The antioxidant activity of clove oil was assessed by means of DPPH. The CEO proved to be a strong antioxidant, mainly due to its high eugenol content, yielding an IC_50_ value of 158 µg/mL, but did not outperform ascorbic acid nor butylated hydroxyanisole. These results are comparable to the literature reports, with a wide range of DPPH-derived IC_50_ values available. For example, Martiniaková et al. reported a 0.82 ± 0.02 mg/ML IC_50_ value for their tested commercially available clove oil [36]. This value is five times higher than the value reported herein. Nevertheless, these numbers are expected to vary, as phenolic content varies among oils extracted from the same species by the same method. Alfikri et al. suggested that antioxidant activity varies between oils extracted from the same species at different growth stages. This particular work assessed the yield, quality, and antioxidant activity of Zanzibar clove (*Syzygium aromaticum* L.) bud oil between three phenological stages in young (3–4 years) and mature trees (45 years). The group reported DPPH scavenging activity IC_50_ values ranging between 15.80 and 108.85 µg/mL [37]. 

Antioxidants act as defensive tools against the effects of free radicals within the human body, where they eliminate ROS while also adjusting cellular redox state and facilitating the transduction of redox signals [38]; considering that ROS formed as a result of oxidative stress are deemed responsible for the development of numerous chronic diseases including cancer, finding antioxidant agents of natural origin lacking side effects has become a focus of current research. Our results are in line with previous research that showed strong antioxidant effects for clove oil due to the presence of phenolic compounds in its composition [37]. Additionally, when tested in a nematode animal model, the antioxidant activity of clove oil by means of increased expression of SOD-3 or GST-4 led to diminished ROS concentration and prolonged lifespan [39].

The anticancer activity of clove oil was assessed in several cancer cell lines; PEG-400 and Tween 20 were used as emulsifiers in order to increase the dispersion of the lipophilic essential oil within the aqueous cell culture medium. PEG 400 and Tween 20 were chosen as emulsifying agents due to their stability, lack of toxicity, and water solubility properties [40,41]. 

Our results showed that in most cancer cell lines, PEG and Tween CEO formulations exhibited stronger cytotoxic effects than the simple CEO water dispersion, presumably due to higher amounts of active compounds transported in the aqueous phase through the hydrophilic carrier; in all cases, the cytotoxic activity manifested in a dose-dependent manner and displayed selective character, as showed by the lack of anti-proliferative effects against human keratinocytes HaCaT. Moreover, in all cell lines, PEG and Tween CEO formulations increased LDH release, thus suggesting the compounds’ ability to selectively cause cellular membrane damage. When tested in RPMI-7951 melanoma cells, all concentrations induced stronger effects compared to eugenol except for the simple 0.5% water dispersion; the effect of eugenol against nine melanoma cell lines was tested by Pisano et al., who reported relatively poor cytotoxic activity (around 80% cell viability) except for the 13,443 cells where the cellular viability dropped to 60% [42]. In our experiment, the RPMI-7951 melanoma cells exhibited less than 50% cell viability after eugenol treatment and even less (~25%) following treatment with CEO samples, indicating strong cytotoxic activity; a comparable inhibitory activity was reported for the essential oil prepared from clove buds in B16 melanoma cells when similar concentrations were used [43]. A strong cytotoxic effect was reported for clove essential oil in A375 melanoma cells but without selectivity, which makes the clinical application difficult [44]. To the best of our knowledge, this is the first study in RPMI-7951 melanoma cells that exhibit epithelial morphology and a potent angiogenic effect not found in other melanoma cell lines that correlate with its metastatic potential [45].

In HT-29 cancer cells, eugenol induces a 50% reduction in cell viability and increased with 40% LDH release, and clove essential oil samples exhibited stronger cytotoxic effects but only when applied in higher concentrations; interestingly, the simple CEO water dispersion acted more effectively than the corresponding PEG-400 dispersions. Clove oil was also tested in Caco-2 colon cancer cells, where it increased programmed cell death and inhibited proliferation signaling by modulating Raf-1 activity [15]; in HT-29 cells, a nanoparticulate system containing clove oil inhibited cell proliferation in a selective manner [46]. In a study published in 2013, the ethyl acetate extract of clove buds was tested in HT-29 xenografts in vivo, where it suppressed tumor growth; however, oleanolic acid was identified as responsible for such antitumor activity, and the compound was not detected in our CEO sample [16]. Abadi et al. have examined the antiproliferative effects in HT-29 cells of clove essential oil nanoemulsions obtained using Tween 20, 80, and PEG [46]; the nanoemulsions were able to inhibit the proliferation of cancer cells in a time- and dose-dependent manner and induce apoptosis by upregulating caspase-3 activity. The in vivo experiments revealed that the clove oil nanoemulsions did not cause cell death in mice liver, kidney, and jejunum; they may even be suitable for suppressing the side effects associated with certain cytostatic drugs due to their cytoprotective effects in mice liver cells. Taking into consideration that eugenol is the main compound found in clove essential oil and that eugenol was found very active in HT-29 cancer cells with an IC_50_ of 500 µM [47], we may safely assume that eugenol is responsible for the antiproliferative activity emphasized by our study. However, the stronger cytotoxic activity recorded for the essential oil samples versus eugenol can be attributed to their complex structures characterized by plurality and diversity of chemical components that support interactive effects able to modulate biochemical pathways and therapeutic targets such as membrane potential and receptor selectivity [48].

In A431 skin cancer cells, significant cytotoxic effects occurred for all samples; however, stronger effects compared to eugenol were only obtained through the application of surfactant-based CEO water dispersions in higher concentrations (200 µg/mL), which increased LDH release to 50–60%. A431 cells are human epidermoid carcinoma cells frequently used for the study of skin cancer development as well as for the testing of new therapies against epidermoid carcinoma that can be triggered by UV exposure [49]. The activity of eugenol and clove oil against A431 cells was not previously reported; however, eugenol was able to inhibit the development of DMBA croton oil-induced skin cancer [50], therefore explaining the current findings.

In NCI-H460 large cell lung cancer cells, which exhibit fast growth, eugenol induced strong cytotoxic effects, while the essential clove oil was not able to exceed; however, the essential oil samples also induced significant antiproliferative effects, as showed by the low values of cell viability (around 60%) and increased LDH release (around 45%). A chloroform extract of clove was revealed as an effective anticancer agent against two lung cancer cell lines, A549 and H1299 [51]; eugenol and clove oil exerted cytotoxic effects against A549 lung cancer cells [52]. However, the data on anticancer activity of both eugenol and clove oil in lung cancer cells are still scarce; as an example, a sub-fraction of a methanolic extract of clove buds containing eugenol was found active against NCI-H460 cells with the anticancer activity attributed to eugenol [53]. Further studies are therefore needed to elucidate both the effectiveness of clove oil as an anticancer agent and the underlying molecular mechanisms.

As previously mentioned, none of the tested samples exerted significant cytotoxic effects against non-cancerous HaCaT cells (human keratinocytes), thus revealing selective anticancer effects against all tested cell lines. 

The role of ROS in cancer development, progression, and metastasis has been extensively studied in the last decades. Compared to normal cells, cancer cells present a higher basal ROS production that is vital to ensure cancer cell homeostasis and survival and to facilitate differentiation, proliferation, and migration [54]. Hence, strategies aimed at lowering ROS production below levels required for cancer cell survival or strategies aimed at significantly increasing ROS levels, in order to overwhelm the redox adaptation mechanisms and to induce cancer cell death, are promising approaches currently investigated in the development of more effective anticancer agents [54,55]. In this context, eugenol has been described as a dual agent, having both antioxidant and pro-oxidant effects on cancer cells. The review of Bezzera et al. presents numerous studies whose results support the role of eugenol in both the prevention of cancer formation and cancer treatment, considering its relationship with its antioxidant and pro-oxidant activities [56]. There are studies that suggest that eugenol has a cytotoxic effect in the μM range on various melanoma, leukemia, histiocytic lymphoma, hepatocellular, breast adenocarcinoma, lung, and cervix carcinoma cell lines that is accompanied by increased ROS production [57,58]. Similarly, in HCT-15 colon cancer cells, 48 h treatment with eugenol increased almost threefold ROS generation; this event was followed by DNA fragmentation, increased p53 activation PARP cleavage, and apoptosis [59]. In contrast, the group of Atsumi et al. reports a cytotoxic effect in the mM range against oral squamous cell carcinoma (HSC-2) and submandibular gland adenocarcinoma (HSC) cell lines; however, no changes in ROS production were reported [60,61]. Our results show that eugenol was able to increase ROS production in RPMI-7951, HT-29, A431, and NCI-H460 cell lines. Moreover, the CEO formulations with PEG-400 and Tween-20 increased ROS production in all the tested cancer cell lines; the increases in ROS levels were higher than those induced by eugenol alone in RPMI-7951, HT-29, and A431 cell lines. Although the antioxidant effects are well known, eugenol/CEO can act as pro-oxidants under specific conditions, such as in the presence of high ROS levels, as seen in cancer cells. As demonstrated, in normal fibroblast (NIH/3T3) and embryonic kidney (HEK-293) cell lines, treatment with 100 μg/mL eugenol was found to decrease ROS levels while increasing the cell resistance to H_2_O_2_ [62]. The shift to towards ROS production can be explained by assessing the function of the primary ROS generation site: the mitochondria; these organelles are known to generate nearly 90% of ROS, mainly at the level of complex I (CI) and complex III (CIII) of the electron transport chain (ETC) [63]. 

Given the crucial role of mitochondria in ROS production, this study also investigated the effect of eugenol and CEO on the mitochondrial function of various cancer cells. After a thorough literature review, and to the best of our knowledge, the present study is the first that specifically investigates the effects of eugenol 100 μg/mL and CEO 1% on RPMI-7951, HT-29, A431, and NCI-H460 cancer cell lines using high-resolution respirometry. During the experimental protocol, digitonin was used to selectively permeabilize the cell membrane. The optimal digitonin concentration was determined after a stepwise titration protocol for each cell line, as described by Pesta and Gnaiger [64]; at this optimal concentration, digitonin does not affect the mitochondrial membrane and allows researchers to use various substrates and inhibitors to effectively assess mitochondrial respiration. Upon evaluating each of the obtained mitochondrial respiratory rates, we observed that both eugenol and CEO were able to significantly decrease the active respiration dependent on CI and on CI+CII (OXPHOS_CI_ and OXPHOS_CI+II_), thus suggesting that both compounds can inhibit the oxidative phosphorylation. This effect was observed in all cell lines used in this study and was in accordance with the cell viability results, thus suggesting a correlation between the cytotoxic effect and mitochondrial inhibition. However, the degree of inhibition varied based on the cell lines, the compounds, and the substrates used. Specifically, the strongest inhibition was observed for CEO in RPMI-7951 and HT-29 cells, whereas eugenol was more potent in inhibiting the mitochondrial respiration of A431 and NCI-H460 cancer cells. Also, the inhibitory effects on active respiration were stronger when only CI substrates were added (OXPHOS_CI_) compared with the active respiration dependent on both CI and CII substrates (OXPHOS_CI+II_). These results suggest that the inhibitory mechanism of both eugenol and CEO is independent on CII substrates. When analyzing State 4, we observed that eugenol and CEO increased this respiratory rate compared to control. This effect was firstly described by Terada in 1981 [65], who reported the impact of uncouplers on mitochondrial respiration. Accordingly, uncouplers are agents, typically small and hydrophobic molecules, that can dissipate the mitochondrial proton gradient (ΔpH) and membrane potential (ΔΨm) by increasing the proton transfer across the inner mitochondrial membrane (IMM) but not through complex V (ATP-synthase). This process ultimately leads to the decoupling of substrate oxidation from ADP phosphorylation, increased oxygen consumption, and reduced ATP production [65]. In our case, by increasing State 4, both eugenol and CEO acted as uncoupling agents that increase the proton leak across the IMM. Cotmore et al. reported a similar effect of eugenol on isolated rat liver mitochondria [66]. More precisely, the study showed that eugenol significantly decreases, at low doses (0.22–0.88 mM) and in a dose-dependent manner, State 3 respiration (OXPHOS) while also acting as an uncoupling agent at high concentrations (0.88–3.5 mM). Similarly, another study demonstrated that eugenol has an inhibitory effect on mitochondrial respiration of SCC-4 human squamous cell carcinoma [67]. Normally, the ETS rate is obtained after successive titrations with FCCP, a classical protonophore that transports protons across the IMM and dissipates the electrochemical ΔpH, thus yielding an estimate of maximal electron transport capacity [68] (polarographic oxygen sensors). When adding an uncoupling agent, one would expect a further change in the ETS capacity and also an increase in State 2 respiratory rate [64,69]. However, in our study, the addition of either eugenol or CEO did not modify the ETS or State 2. These results can be explained by reviewing the limited literature; several years after the study of Cotmore et al., the group of Usta et al. demonstrated in the same in vitro rat liver mitochondria model that eugenol inhibits NADH oxidase (CI) and reduces the ΔΨm and ATP production without having any effect on succinate dehydrogenase activity (CII) [70]. The inhibitory effect of eugenol on CI taken together with the uncoupling effect explains why we did not record any significant changes in State 2 or ETS upon treatment with eugenol or CEO. The ETS was unaffected by CI inhibition due to the presence of an active CII that allowed an independent electron flow. Normally, when CI is inhibited, State 2 respiratory rate is lower compared to normal conditions where CI is functioning [71,72]. Due to their inhibitory effect on CI, this result should have been observed in our case upon treatment with eugenol and CEO; nonetheless, in our case, the decrease in State 2 was not observed, probably due to the parallel uncoupling effect of these compounds that may bypass the inhibited CI through other parts of the electron transport chain. This induced mitochondrial dysfunction may itself lead to increased ROS production [73]. More precisely, studies have shown that a CI blockade increases ROS production and reduces mitochondrial respiration [74]. Moreover, even though the mild mitochondrial uncoupling is considered to be a mechanism to prevent the excessive accumulation of ROS in mitochondria by decreasing its production, an excessive uncoupling can induce ROS production; researchers reported that classic uncouplers such as FCCP and 2,4-Dinitrophenol (DNP) are indeed able to increase ROS production in pulmonary adenocarcinoma (Calu-6), juxtaglomerular carcinoma (As4.1), T-cell leukemia (Jurkat), lymphoblastic leukemia (CEM), and human cervical cancer (HeLa) cell lines [75,76,77,78,79]. Hence, taken together, the inhibition of mitochondrial respiration and the uncoupling effect of eugenol and CEO may explain the increase in ROS production observed in the treated cell lines. 

To extrapolate CEO’s use to a possible in vivo experimental stage, we employed in silico-based techniques to anticipate possible issues that would prevent CEO from being further researched. Two main predictions were possible concerns. The SwissADME server predicted that a large part of the components may inhibit various isoforms of CYP450. This would indeed pose as a problem if this oil were administered together with other therapeutically active compounds that were to be metabolized by the same enzymes. However, it could also be an advantage if one compound would inhibit the enzyme responsible for the inactivation of other CEO components, thus increasing their biological activity by impairing their metabolism. These predictions are in line with the findings of Alharbi et. al, which showed that eugenol, the main component of CEO, inhibits by various degrees and in a dose-dependent manner CYP450 isoforms such as CYP1A2, CYP2C9, CYP2D6, and CYP3A4 [80]. 

Secondly, the PreADMET server predicted that all CEO components might induce carcinogenicity in rats, mice, or both models. According to their website [23], the model based on which rodent carcinogenicity is predicted is based on two years of in vivo exposure results from NTP and FDA. This model does not seem to include a dose-based exposure as well. Having said that, it is hard to claim that the ten CEO components are potentially carcinogenic. The main component, eugenol, is the only CEO constituent that is classified by the International Agency for Research on Cancer (IARC) as non-carcinogenic [81], while the rest of the nine constituents are not even classified by IARC, probably due to a lack of concluding toxicological evidence. Therefore, we can conclude that there may be a risk associated with these compounds; however, this risk is dose-dependent and time-dependent and does not pose an inconvenience to the research potential of CEO.

## 4. Materials and Methods

### 4.1. CEO Extraction and GC-MS Analysis

Dried plant material (cloves) was received from the University of Life Sciences “King Michael I” from Timisoara Romania Herbarium with the voucher number VSNH.BUASTM–127. Dried cloves were ground, after which 100 g of dried clove powder was subjected to steam hydrodistillation (4 h, 100 °C) using a Craveiro-type apparatus (The resulted steam was condensed and collected in a water-cooled oil receiver to decrease the formation of artifacts due to overheating (10.3390/foods10040815). After separation, the oil was dried using anhydrous sodium sulfate and stored at −18 °C in sealed vials for future analysis. The extraction yield was calculated as the ratio of obtained oil weight/dried plant weight × 100 (wt%). 

A GC Hewlett Packard HP 6890 Series gas chromatograph (Agilent, Santa Clara, CA, USA) coupled with a Hewlett Packard 5973 Mass Selective Detector was used for analysis. A total of 1 μL of diluted sample (1:100 in hexane) was injected following the GC-MS operation conditions: DB-WAX capillary column (30 m length, 0.25 mm internal diameter, 0.25 μm film thickness), oven temperature range between 50 °C to 250 °C with 6 °C/min, and a solvent delay of 4 min. The MS source was set at 230 °C, and the MS Quad was set at 150 °C. The helium gas flow which leads the sample through the column was at a rate of 1 mL/min. The scanned compounds were in the range of mass between 50 to 600 amu. All compounds were evaluated based on their spectra compared to the mass spectrum from the NIST2.0 library (USA National Institute of Science and Technology software, Gaithesburg, MD, USA), and area percentage was established. A qualitative quantitation by calculating retention indexes based on the retention times and areas of C9 to C18 alkanes was performed; also, the Adams Indexes were shown for comparation with the literature.

### 4.2. DPPH Antioxidant Scavenging Activity Determination 

To evaluate the antioxidant scavenging activity, the 2,2-diphenyl-1-picrylhydrazyl (DPPH) radical assay was applied following the methodology described by Rădulescu et al. [82]. A 5 mg DPPH was prepared in ethanol (5 mL), from which further dilutions were made to generate a calibration curve with concentrations ranging from 7.81 µg/mL to 0.5 mg/mL. Positive controls, ascorbic acid (AA) and butylated hydroxyanisole (BHA), were diluted in serial concentrations ranging from 0.06 µg/mL to 1.3 mg/mL. A plant 1:10 ethanolic dilution of the obtained oil extract was mixed in a ratio of 1:4 (*v*/*v*) with 0.25 mM DPPH ethanolic solution and incubated in the dark at 25 °C. Absorbance was measured at 515 nm using a Tecan Infinite 200Pro spectrophotometer (Tecan Group Ltd., Männedorf, Switzerland, i-control software version 1.10.4.0).

The inhibition percentage (Inh%) was calculated using the formula:

Inh% = ((A_0_ − A_s_)/A_0_) × 100

where A_0_ represents the absorbance of the control and A_s_ represents the absorbance of the sample. The inhibition values were further used to calculate the IC_50_, resulting from the calibration curve equation specific to each sample and control, with the results expressed as Inh% relative to concentration (µg/mL).

### 4.3. Cell Culture 

Human keratinocytes HaCaT (purchased from CLS Cell Lines Service GmbH, Eppelheim, Germany), malignant melanoma RPMI-7951, colorectal adenocarcinoma HT-29, skin carcinoma A431, and non-small lung NCI-H460 human cancer cells (all purchased from American type Culture Collection, ATTC, Lomianki, Poland) were selected for our study. All cell lines were cultured in their specific cell culture media, supplemented with 10% fetal bovine serum and 1% penicillin/streptomycin, following the guidelines provided by the manufacturer. The cells were maintained in a humidified incubator at 5% CO_2_ at 37 °C.

### 4.4. Cell Viability Assessment

The Alamar blue colorimetric assay was employed for evaluating cell viability of HaCaT, RPMI-7951, HT-29, A431, and NCI-H460 cells after 48 h stimulation with 0.5% and 1% CEO and with 100 μg/mL and 200 μg/mL CEO dispersed in PEG 400 and Tween 20, respectively. The cells (1 × 10^4^/well) were seeded onto 96-well plates and incubated at 37 °C with 5% CO_2_ until reaching 80–85% confluence. Afterwards, the used media was discarded, and the cells were stimulated with fresh media and the tested concentrations of CEO. After 48 h, the cells were counterstained using 0.01% Alamar blue and incubated for another 3 h. The absorbance measurements were determined at 570/600 nm using an xMark™ Microplate Spectrophotometer, Bio-Rad (Hercules, CA, USA). The experiments were performed in triplicate.

### 4.5. Lactate Dehydrogenase (LDH) Release Quantification

The cytotoxic potential of eugenol (100 μg/mL), CEO (0.5%, 1%), CEO-PEG (100 and 200 μg/mL), and CEO-Tw (100 and 200 μg/mL) on normal HaCaT cells and RPMI-7951, HT-29, A341, and NCI-H460 cancer cells was evaluated after 48 h of treatment by quantifying the extracellular release of the cytosolic LDH in the culture medium. The cells were seeded in 96-well plates (1 × 10^4^ cells/well), incubated until they reached appropriate confluence, and treated with the tested compounds for 48 h. After the treatment period, 50 μL of culture medium was collected from each well, transferred to a new 96-well plate together with 50 µL of LDH reaction mixture, and incubated for 30 min at room temperature. The reaction was stopped with 50 µL of stop solution. The absorbance was read at 490 nm and 680 nm using an xMark™ Microplate Spectrophotometer, Bio-Rad (Hercules, CA, USA). 

### 4.6. Assessement of Celullar ROS Production

The ROS production in RPMI-7951, HT-29, A341, and NCI-H460 cells treated for 48 h with eugenol (100 μg/mL), CEO (1%), CEO-PEG (200 μg/mL), and CEO-Tw (200 μg/mL) was evaluated using the cell-permeant 2′,7′-dichlorodihydrofluorescein diacetate (H_2_DCFDA) kit (ab113851, Abcam, Cambridge, UK). The detection method is based on the ability of the nonfluorescent H_2_DCFDA to convert to highly fluorescent 2′,7′-dichlorofluorescein (DCF) when cleaved by ROS. ROS production was quantified at Ex/Em: 485/535 nm using the BioTek Synergy HTX multimode microplate reader (Agilent Technologies, Santa Clara, CA, USA), following the protocol described by the manufacturer [83]. 

### 4.7. High-Resolution Respirometry

The mitochondrial function was assessed at 37 °C using the high-resolution respirometer Oroboros (Oxygraph-2k Oroboros Instruments GmbH, Innsbruck, Austria). The mitochondrial respiratory rates were obtained using a modified substrate uncoupler-inhibitor-titration (SUIT) protocol, as previously described by Petruș et al. [84]. The cells used in this study, RPMI-7951, HT-29, A341, and NCI-H460, were cultured in T25 culture flasks until reaching 80–85% confluence, trypsinized, counted, and resuspend (1 × 10^6^/mL cells) in a special mitochondrial respiration medium (EGTA 0.5 mM, 3 mM KH_2_PO_4_, taurine 20 mM, K-lactobionate 60 mM, MgCl_2_ 10 mM, D-sucrose 110 mM, HEPES 20 mM, and BSA 1 g/L, pH 7.1). The oxygen flux was allowed to stabilize for 15 min after the cells were added to the chambers of the device. The first step in the protocol was the addition of digitonin (20–35 μg/L × 10^6^ cells), glutamate (10 mM), and malate (5 mM); digitonin was used to permeabilize the cell membrane, while the complex I (CI) substrates addition, glutamate and malate, allowed the measurement of basal respiration dependent on endogenous ADP, also known as State 2. The next step was ADP (5 mM) addition that enabled the measurement of the active respiration dependent on CI (OXPHOS_CI_); this was followed by the addition of a complex II (CII) substrate, succinate (10 mM), which enabled the measurement of the active respiration dependent on both CI and CII (OXPHOS_CI+II_). Subsequently, complex V was inhibited with oligomycin (1 μg/mL) resulting in the measurement of leak respiration dependent on CI and CII (State 4). Afterwards, P-(trifluoromethoxy) phenylhydrazone carbonyl cyanide-FCCP was titrated in steps (1 μM/step) to obtain and measure the maximal respiratory capacity of the electron transport system (ETS_CI+II_). Rotenone (0.5 μM, a CI inhibitor) was added to obtain the ETS dependent solely on CI. In the final step of the protocol, an inhibitor of CIII, antimycin A (2.5 μM), was added to inhibit the mitochondrial respiration and to measure the residual oxygen consumption (ROX). The final values were obtained after ROX correction.

### 4.8. Statistical Analysis 

The results obtained in cell viability and cytotoxicity assays, as well as the results obtained after the evaluation of ROS production, were analyzed statistically using one-way ANOVA followed by the Dunnett post-test. The statistical differences vs. control, for the high-resolution respirometry studies, were determined using two-way ANOVA with Bonferroni’s multiple comparison post-test. All the differences were considered to be statistically significant if *p* < 0.05 (* *p* < 0.05, ** *p* < 0.01, and *** *p* < 0.001).

### 4.9. Molecular Docking 

Molecular docking was employed using a previously described workflow [85]. Briefly, all protein target structures, obtained from the RCSB Protein Data Bank [86], were optimized as suitable docking targets, with Autodock Tools v1.5.6 (The Scripps Research Institute, La Jolla, CA, USA) (Table 4). The SDF structure files for the ten CEO components were retrieved from PubChem [87] and were converted into 3D structures using PyRx’s Open Babel module [88]. Molecular docking was achieved with PyRx v0.8 [88] virtual screening software (The Scripps Research Institute, La Jolla, CA, USA) using Vina’s encoded scoring function [89]. The docking protocol was validated by re-docking the native ligands into their original protein binding sites. The root means square deviation (RMSD) between the predicted and experimental docking pose of the native ligand was calculated. Molecular docking was performed only for cases with aforementioned RMSD values not exceeding a 2 Å threshold. The docking grid box coordinates and size were selected to best fit the active binding site (Table 7). Docking scores were recorded as ΔG binding energy values (kcal/mol). Protein–ligand binding interactions were analyzed using Accelrys Discovery Studio Visualizer 4.1 (Dassault Systems BIOVIA, San Diego, CA, USA).

### 4.10. In Silico ADMET Predictions for the CEO Components

In this work, we assessed various theoretical characteristics regarding the pharmacokinetic and toxicologic properties of the ten CEO components. These predictions were achieved using the PreADMET [23] and SwissADME [98] servers. For the SwissADME predictions, each CEO component was submitted as a SMILE string based on which the server generated ADME and other useful predicted parameters. The PreADMET server was used to retrieve toxicology-predicted data for the CEO components based on their submitted 2D structure. 

## 5. Conclusions

Our study concludes that clove essential oil, especially when formulated with solubilizing agents such as Tween-20 or PEG-400, acts as a selective cytotoxic substance against melanoma (RPMI-7951) and colorectal adenocarcinoma cells (HT-29). This study suggests that the cytotoxic activity of CEO is mainly attributed to its high eugenol contents. However, in some cases, this cytotoxic activity is enhanced by the presence of other minor constituents that may synergistically affect the cancer cells’ viability. This selective action was also present when CEO disrupted mitochondrial function in the aforementioned cell lines but did not significantly affect normal cells. This study reports for the first time the effects of CEO, and its formulations with Tween-20 and PEG-400, on the mitochondrial function of RPMI-7951, HT-29, A431, and NCI-H460 cancer cell lines, thus providing novel insights into their impact on cancer cell bioenergetics. Moreover, this study is the first to report a molecular docking-based workflow meant to investigate the interactions of the ten components of CEO with key druggable protein targets—EGFR1, VEGFR2, PI3Kα, MEK1, AKT/PKB, mTOR, Bcl-XL, and Bcl-2—associated with cancer cell proliferation and survival, thus providing valuable insights into the potential protein-targeted mechanisms underlying the observed anticancer activity. Overall, based on the evidence shown, the present study reiterates that CEO could be an effective and safer alternative/complementary option for cancer treatment, thus condoning further research in this field.

## Figures and Tables

**Table 1:** Chemical composition of CEO determined through GC-MS.

No	Compound Name	RT	RI Calc	AI	Area % Calc
**1**	Copaene	11.74	1299	1376	0.38
**2**	β-Caryophyllene	13.88	1411	1418	20.22
**3**	α-Caryophyllene (Humulene)	15.22	1482		2.84
**4**	α-Cubebene	16.82	1567	1387	0.34
**5**	Calamenene	18.18	1638	1528	0.24
**6**	2,5,9-Trimethylcycloundeca-4,8-dienone	20.02	1735		0.11
**7**	Caryophyllene oxide	20.85	1779	1582	0.11
**8**	1,2-Dimethylcyclohexane	21.07	1791		0.22
**9**	3-cyclohexen-1-carboxaldehyde,3,4-dimethyl- (3,4-Dimethyl-3-cyclohexenylmethanal)	21.78	1828		0.10
**10**	Eugenol	23.88	1939	1356	75.26
**Total**					99.82

**Table 2:** DPPH scavenging antioxidant activity of CEO expressed as IC50 value (µg/mL).

Sample	IC_50_ (µg/mL)
**CEO**	158.07 ± 0.52
**AA**	11.66 ± 1.27
**BHA**	35.13 ± 0.32

**Table 3:** The mean mitochondrial respiratory rates (pmol/(s × million cells)) after the treatment of RPMI-7951, HT-29, A431 and NCI-H460 cells with eugenol and CEO.

		State 2	OXPHOS_CI_	OXPHOS_CI+II_	State 4	ETS_CI+II_	ETS_CI_
*RPMI-7951*	Control	4.49	22.66	34.83	5.70	42.46	28.31
	Eugenol	8.313	13.74 *	26.84 *	15.21 *	45.07	30.33
	CEO	7.57	11.74 **	23.75 **	19.12 ***	45.26	32.37
*HT-29*	Control	4.81	32.02	46.37	5.18	55.89	25.07
	Eugenol	7.98	20.53 **	36.23 *	13.79 **	59.64	31.07
	CEO	8.87	17.49 ***	31.69 **	16.05 ***	63.35	31.94
*A431*	Control	4.42	27.19	44.10	8.82	45.83	32.67
	Eugenol	4.04	14.63 ***	29.22 **	18.46 **	45.24	58.29
	CEO	5.55	17.51 **	34.75 *	15.60 *	45.40	36.01
*NCI-H460*	Control	11.89	36.38	54.64	18.29	69.60	40.23
	Eugenol	11.45	24.32 ***	43.90 ***	26.84 **	69.27	41.66
	CEO	10.76	20.34 ***	34.54 **	28.59 *	68.13	41.19

* *p* < 0.05, ** *p* < 0.01, and *** *p* < 0.001.

**Table 4:** The docking scores of compounds **1**–**10**.

Compound	Protein Targets							
	EGFR1	VEGFR2	MEK1	AKT/PKB	PI3Kα	MTOR	BCL-2	BCL-XL
	Binding Affinity (kcal/mol)							
**NL**	**−10.9**	**−12**	**−9.4**	**−9.4**	**−8.8**	**−11.2**	−10.7	−10.7
**1**	−7.2	−7	−6.7	−6.6	−7.5	−6.5	−7.4	−7.5
**2**	−7.2	−7.1	−6.6	−6.2	−6.6	−6.8	−6.9	−7
**3**	−7	−6.5	−6.9	−6.3	−6.2	−6.3	−6.8	−6.8
**4**	−7.5	−7	−6.8	−6.6	−7.2	−6.8	−6.8	−7.2
**5**	−7.7	−7.4	−7	−7.3	−7.5	−7.3	−7	−7.2
**6**	−7.4	−7.2	−6.8	−7	−7.1	−6.9	−7	−7
**7**	−7.1	−7	−7.2	−6.3	−6.4	−6.3	−6.9	−6.9
**8**	−4.8	−5.4	−5.3	−5.3	−4.9	−4.9	−5.2	−5.5
**9**	−5.9	−5.9	−5.7	−5.8	−5.7	−5.8	−5.3	−5.7
**10**	−6	−6.7	−5.8	−6.5	−5.9	−5.9	−5.6	−5.4

**Table 5:** SwissADME predicted pharmacokinetic properties for the 10 CEO components.

Compound ID	GI Absorption	BBB Permeant	Pgp Substrate	CYP1A2 Inhibitor	CYP2C19 Inhibitor	CYP2C9 Inhibitor	CYP2D6 Inhibitor	CYP3A4 Inhibitor
**1**	Low	Yes	No	Yes	Yes	Yes	No	No
**2**	Low	No	No	No	Yes	Yes	No	No
**3**	Low	No	No	No	No	Yes	No	No
**4**	Low	Yes	No	Yes	Yes	Yes	No	No
**5**	Low	No	No	No	No	No	Yes	No
**6**	High	Yes	No	No	No	No	No	No
**7**	High	Yes	No	No	Yes	Yes	No	No
**8**	Low	Yes	No	No	No	No	No	No
**9**	High	Yes	No	No	No	No	No	No
**10**	High	Yes	No	Yes	No	No	No	No

**Table 6:** PreADMET predicted toxicologic properties for the 10 CEO components.

Compound	Carcino_Mouse	Carcino_Rat	Daphnia_at (mg/L)	hERG_Inhibition	Medaka_at (mg/L)	Minnow_at (mg/L)
**1**	negative	positive	0.151468	medium_risk	0.0264343	0.00859689
**2**	negative	positive	0.0194637	medium_risk	0.0005590	0.000587276
**3**	positive	positive	0.0868411	medium_risk	0.0092247	0.00235616
**4**	negative	positive	0.151468	medium_risk	0.0264343	0.0086039
**5**	positive	negative	0.0778505	medium_risk	0.0075105	0.00223027
**6**	positive	positive	0.139787	low_risk	0.0229798	0.00620924
**7**	positive	positive	0.0794482	medium_risk	0.0083202	
**8**	negative	positive	0.262287	medium_risk	0.0679829	0.0612808
**9**	negative	positive	0.443932	low_risk	0.204963	0.160983
**10**	positive	positive	0.118703	medium_risk	0.0188822	0.0124586

**Table 7:** Docking parameters used for the in silico evaluation of the CEO components.

Protein (PDB ID)	Grid Box Center Coordinates	Grid Box Size	Native Ligand	References
**EGFR1 (1XKK)**	x = 17.6841 y = 32.5941 z = 36.1957	x = 17.8102 y = 15.3373 z = 20.8923	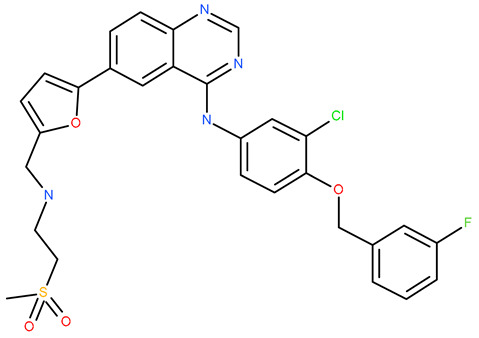 Lapatinib	[90]
**VEGFR2 (4ASD)**	x = −23.4657 y = −0.9992 z = −11.3508	x = 18.4692 y = 15.7547 z = 16.5252	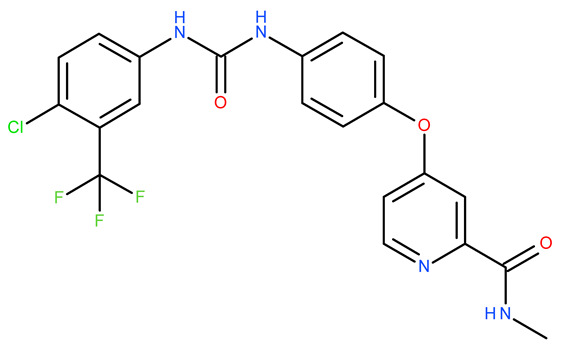 Sorafenib	[91]
**MEK1** **(3DV3)**	x = 38.1030 y = −13.4921 z = 0.4310	x = 15.1913 y = 15.1913 z = 16.0921	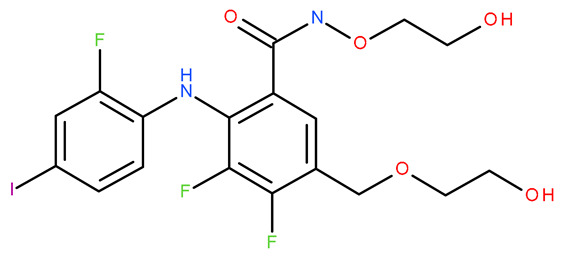 3,4-difluoro-2-[(2-fluoro-4-iodophenyl)amino]-N-(2-hydroxyethoxy)-5-[(2-hydroxyethoxy)methyl]benzamide	[92]
**AKT/PKB** **(4GV1)**	x = −19.9622 y = 3.2356 z = 11.9128	x = 14.0395 y = 10.8823 z = 14.5507	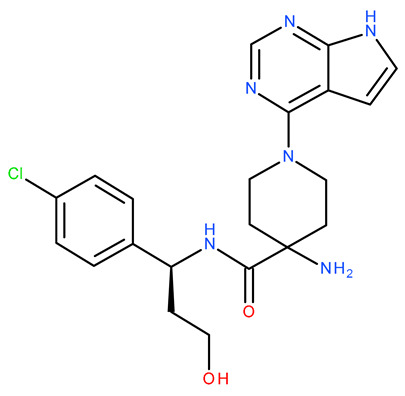 Capivasertib	[93]
**PI3Kα** **(6GVF)**	x = −17.2531 y = 146.3754 z = 27.0137	x = 11.5423 y = 12.3408 z = 14.8070	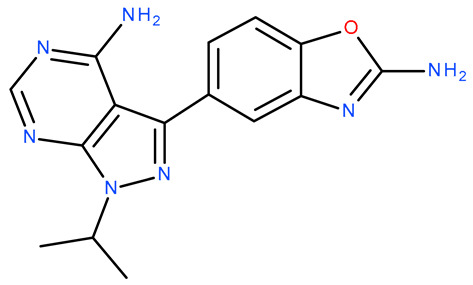 Sapanisertib	[94]
**mTOR** **(4JSX)**	x = 50.3620 y = −1.3027 z = −47.7442	x = 13.0752 y = 8.8140 z = 12.9457	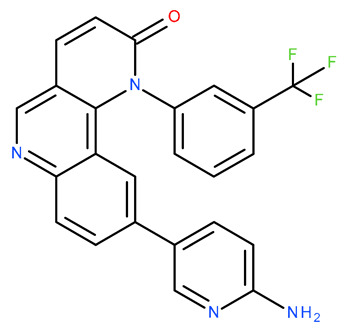 Torin 2	[95]
**BCL-** **2** **(4LVT)**	x = 6.7535 y = −3.7596 z = −8.7819	x = 10.2188 y = 24.8722 z = 14.3830	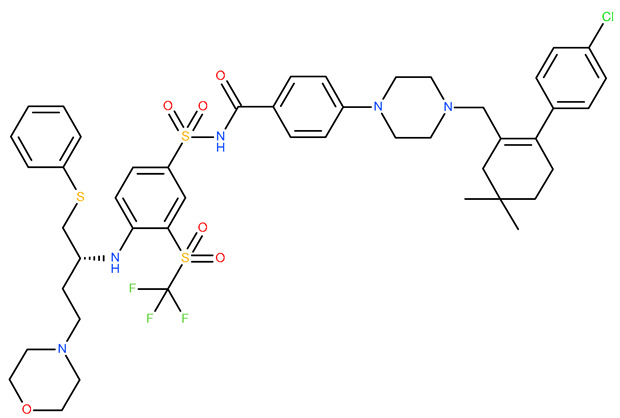 Navitoclax	[96]
**BCL-XL** **(2YXJ)**	x = −8.8537 y = −14.1924 z = 9.8783	x = 15.2788 y = 20.8047 z = 9.7743	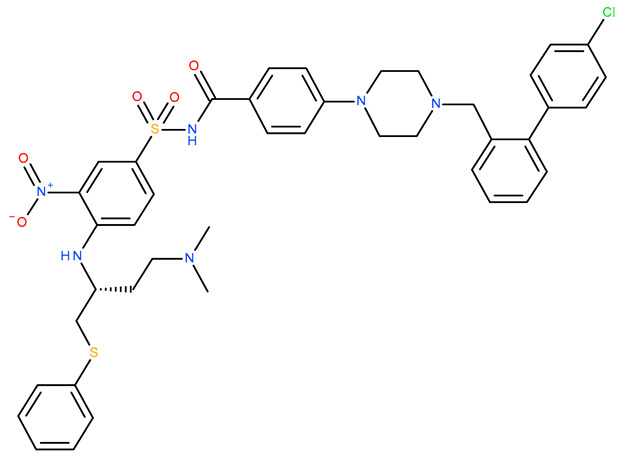 ABT-737	[97]

## Data Availability

The original contributions presented in the study are included in the article, further inquiries can be directed to the corresponding author.
